# Incidence of hydrocephalus in traumatic brain injury: A nationwide population-based cohort study

**DOI:** 10.1097/MD.0000000000017568

**Published:** 2019-10-18

**Authors:** Kai-Hua Chen, Chuan-Pin Lee, Yao-Hsu Yang, Yun-Hsuan Yang, Chien-Min Chen, Mong-Liang Lu, Yi-Chen Lee, Vincent Chin-Hung Chen

**Affiliations:** a Department of Physical Medicine and Rehabilitation, Chang Gung Memorial Hospital, Chiayi; b School of Medicine, Chang Gung University, Taoyuan; c Health Information and Epidemiology Laboratory; d Department of Traditional Chinese Medicine, Chang Gung Memorial Hospital, Chiayi; e School of Traditional Chinese Medicine, College of Medicine, Chang Gung University, Taoyuan; f Department of Pediatric Neurology, Chang Gung Memorial Hospital, Chiayi; g Department of Psychiatry, Wan-Fang Hospital and School of Medicine, College of Medicine, Taipei Medical University, Taipei; h Department of Psychiatry, Chiayi Chang Gung Memorial Hospital, Chiayi, Taiwan.

**Keywords:** brain injuries, hydrocephalus, incidence, propensity score, subarachnoid hemorrhage

## Abstract

**Background:**

The aim of this study was to investigate the risk and peak time of post-traumatic hydrocephalus (PTH) in traumatic brain injury (TBI) patients with traumatic subarachnoid hemorrhage (SAH), compared to TBI patients without traumatic SAH.

**Methods:**

In this retrospective population-based cohort study, the data was extracted from Longitudinal Health Insurance Database from 2000 to 2010 in Taiwan. A total of 23,775 TBI patients who had a first event TBI during 2000 to 2010 were included and divided into TBI with SAH (TBI-S) group and TBI without SAH (TBI-NS) group. We focused on analyzing the PTH in both groups within 2 years after brain injury. Competing risk regression models were performed to assess the risk of developing PTH in the TBI-S group compared to the TBI-NS group.

**Results:**

Comparing to the TBI-NS group, there was a significantly higher cumulative incidence of PTH in the TBI-S group during the 2-year follow-up period. The adjusted hazard ratio (HR) of PTH in TBI-S group within 2 years was between 2.90–3.47, and the highest estimates were obtained within 6 months after injury (HR = 3.47, 95% CI: 2.43–4.94). The occurrence percentage of PTH was highest during 0–3rd month follow-up periods (1.95% in TBI-S group; 0.48% in TBI-NS group).

**Conclusions:**

The peak time of PTH occurrence was noted during 0–3rd month post brain injury. Traumatic SAH patients had an approximate 3-fold risk of developing PTH, comparing to TBI patients without traumatic SAH.

## 1 Introduction

The clinical manifestations of different types of hydrocephalus are varied. In typical clinical symptom of patients with acute high pressure hydrocephalus is increased intracranial pressure (IICP) sign with headache, nausea, vomiting, and papilledema.^[1]^ In idiopathic normal pressure hydrocephalus, the typical clinical triad includes cognitive impairment, gait disturbance, and urine frequency.^[2]^ Post-traumatic hydrocephalus (PTH) is one of the special types of hydrocephalus, which occurs after traumatic brain injury (TBI).^[3]^ However, typical clinical presentations of hydrocephalus in patient with PTH are hard to detect because these symptoms are concealed by underlying sequalae of TBI.^[3]^ During rehabilitation phase, the early sign of PTH is poor improvement, incompatible with injury severity.^[3]^ When the patients are in an unconscious state after TBI, the diagnosis of PTH is more difficult.^[4]^

PTH is a treatable complication of TBI patients.^[4–6]^ A proportion of patients who develop PTH and remain in severe conscious disturbance are benefit from shunt implantation.^[4]^ However, the incidence of PTH is variable, ranging from 0.7% to 45%.^[7–14]^ This wide range of incidence makes physicians hard to decide whether regular brain computed tomography (CT) follow-up as a screening test is necessary. Only in severe pediatric TBI, regular brain CT follow-up is suggested to establish a diagnosis of PTH.^[15]^ A drawback of this approach is the possibility of unnecessary radiation exposure and medical expenditure.^[15]^ The optimal time and the indication for repeating brain imaging remain controversial.^[15]^

Physiologically, cerebrospinal fluid (CSF) is produced by choroid plexus in the ventricular system and then reabsorbed into dural venous sinuses.^[1]^ Under normal circumstance, CSF is in a dynamic balance between production and reabsorption in the ventricular system.^[1]^ After brain injury, this mechanism was interrupted.^[1,6,16]^ In the traditional concept, the mass effect of blood clots within the ventricles preventing CSF flow out of the cranial space is the main cause of acute hydrocephalus.^[1,6]^ Meanwhile, inflammation-mediated adhesions, which obstruct the reuptake of CSF, are the main causes of chronic hydrocephalus.^[16]^ In TBI patients, both mechanisms can lead to the development of PTH.

Although PTH after traumatic subarachnoid hemorrhage (SAH) has been reported back to 1943^[17]^ and further supported by several studies focused on risk factors for PTH following traumatic SAH,^[9,18,19]^ only one study reported on incidence of PTH after traumatic SAH.^[18]^ In contrast, there were numerically more studies focused on spontaneous SAH: its complications,^[20,21]^ treatment^[22]^ and prognosis,^[23]^ and timing of hydrocephalus.^[24]^ It remains unknown, however, whether the foregoing study results can be equally applied to traumatic SAH patients.

For these reasons, further studies about the peak time and high-risk group of PTH are important. Here we evaluate the cumulative incidence, incidence rate, and occurrence percentage of PTH in TBI with traumatic SAH (TBI-S) patient compared to TBI without traumatic SAH (TBI-NS). We additionally evaluate the hazard ratios (HRs) of PTH in TBI-S patients to investigate the risk of PTH in TBI-S patients.

## 2 Materials and methods

### 2.1 General design

In this population-based study, patients who had a first event of hospitalized TBI during 2000 to 2010 were identified from the Longitudinal Health Insurance Database 2005 (LHID2005) in Taiwan. A retrospective cohort analysis was conducted to investigate the risk of PTH during a period of 24 months after TBI-S, compared with TBI-NS patients. As a sensitivity test, we used propensity score matching to control for the baseline conditions and comorbidities for TBI-S and TBI-NS patients at a 1:4 ratio. This study was approved by institutional review board in our hospital (IRB No. 201601634B0).

### 2.2 Introduction of LHID2005

Data for this study were retrieved from the LHID2005 within the period from 1997 to 2013. The National Health Insurance program of Taiwan started in 1995 and enrolls 99% of Taiwanese people. In the LHID2005, the patients’ demographic characteristics, diagnoses, medical expenditures, and prescription claims data were recorded. The diagnostic codes for each patient were based on the clinical modification of the International Classification of Diseases, Ninth Revision, Clinical Modification (*ICD-9-CM*) code. In our study, inpatient claims, emergency, ambulatory care claims, and registry for beneficiaries data of the LHID2005 were used. In this database, the privacy of each patient (name, identification card, medical record number, address, phone number, email, and so on) cannot be recognized. After the approval of institutional review board, the informed consent was not necessary.

### 2.3 Participants

Patients who received a diagnosis of TBI (*ICD-9 CM* code: 800, 801, 802, 803, 804, 850, 851, 852, 853, 854, 959.01) between 1997 and 2010 were identified in inpatient claims data. Only the patients who had the first admission for TBI accompanied with x-ray or CT scan during 2000–2010 were enrolled in this study. The first date of TBI admission was defined as index date. Patients diagnosed with hydrocephalus (*ICD-9 CM* code: 331.3, 331.4) before the index date or patients dying on the admission day were excluded. After exclusion, all TBI patients were divided into TBI-S group and TBI-NS group. To define TBI-S group, TBI patients with *ICD-9 CM* code: 852.0 and 852.1 on the index date were grouped together for analysis. The remaining TBI patients were defined as TBI-NS group.

### 2.4 Main outcome

In clinical practice in Taiwan, the diagnosis of hydrocephalus was based on clinical presentation and radiographic findings. Sometimes, cerebrospinal fluid (CSF) tap test or an external lumbar drainage was performed to increase diagnostic accuracy. In our study, PTH was our main outcome in 2-year follow-up after brain injury. PTH was defined as patients who had a new registration of *ICD-9 CM* code: 331.3 or 331.4 in either the TBI-S group or TBI-NS group 2 years after index date, either at clinic visit or hospital admission. All TBI patients were followed up for no more than 2 years, and patients who died during the follow-up were treated as a competing event against PTH.

### 2.5 Covariates

Comorbid stroke (*ICD-9 CM* code: 431–438), nontraumatic SAH (*ICD-9 CM* code: 430), meningitis (*ICD-9 CM* code: 320, 321, 322), brain tumor (*ICD-9 CM* code: 225, 191,192, 194.3, 194.4), hypertension (*ICD-9 CM* code: 401–405), diabetes mellitus (*ICD-9 CM* code: 250), atrial fibrillation (*ICD-9 CM* code: 427.31), congestive heart failure (*ICD-9 CM* code: 398.91, 402, 404.01, 404.03, 404.11, 404.13, 404.91, 404.93, 428), coronary artery disease (410–413, 414.00, 414.01–414.05) were based on the records before index date, at least 2 clinic visits within 1 year, or 1 hospital admission. All these comorbidities were used as covariates for statistical analysis. The Charlson Comorbidity Index (CCI) score is widely used to measure burden of disease.^[25,26]^ In our study, the CCI score was measured within 1 year before index date.

### 2.6 Statistical analysis

The characteristics of the TBI-S or TBI-NS groups at baseline were summarized as percentages and compared using *χ*^2^ test. Considering underlying comobidities and baseline conditions were different between 2 groups at baseline, these factors may also be the confounding factors of PTH in both groups.^[1,9,18,27,28]^ We simultaneously performed a sensitivity analysis—the propensity score matching—to control these confounders. The probability of causing TBI with/without traumatic SAH (ie, propensity scores) was estimated by a logistic regression on the observed confounders. Using this score, patients in the TBI-S group were matched at a 1:4 ratio to TBI-NS group by a SAS macro.^[29]^

The cumulative incidence of PTH was estimated by using the Kaplan-Meier method for both TBI groups, and comparison was made by the log-rank test. The incidence rate of PTH was calculated as the ratio of the average number of new events per 10,000 person months for TBI-S and TBI-NS groups during each 3-month follow-up periods of 2 years. Risk of PTH between TBI-S or TBI-NS groups was assessed using competing risk regression where death was considered as a competing event.^[30,31]^ The HR of developing PTH during each 3-month follow-up periods of 2 years was calculated. Occurrence percentages of PTH for each group were calculated from new events of PTH divided by at-risk patients in every 3 months. Statistical analyses were performed by one of our authors (Lee CP who is a statistician), using SAS version 9.4 (SAS institute, Cary, NC) and *P* values ≦ 0.05 indicate statistical significance.

## 3 Results

### 3.1 Results of patient selection

**Figure 1:**
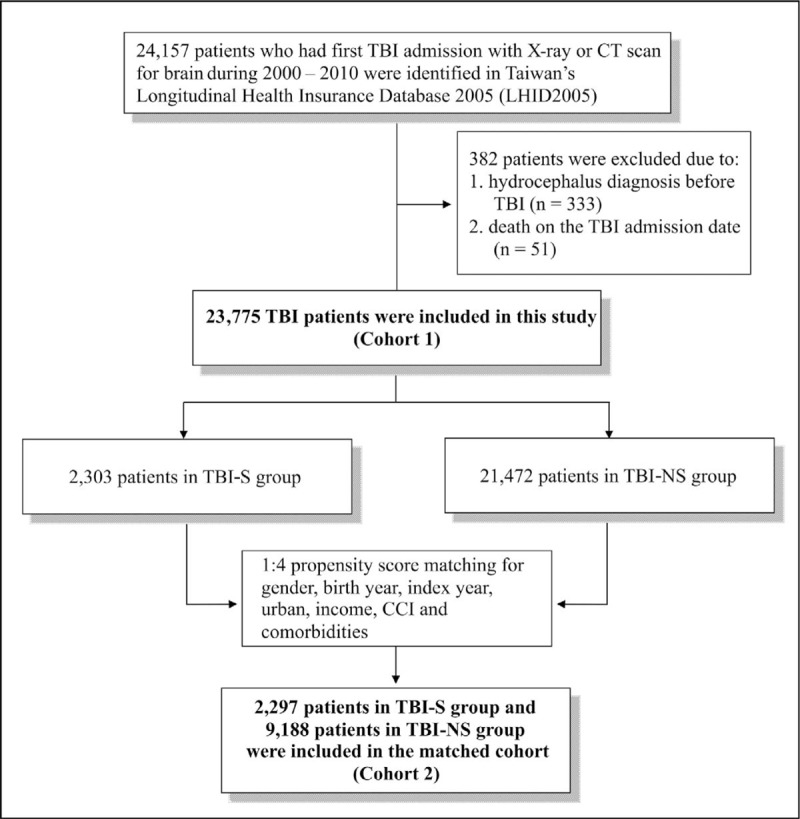
Flow chart of patient selection. CCI = Charlson comorbidity index, TBI = traumatic brain injury, TBI-NS = traumatic brain injury patients without traumatic subarachnoid hemorrhage, TBI-S = traumatic brain injury patients with traumatic subarachnoid hemorrhage.

During 2000 to 2010, there were 24,157 patients with a first event of TBI (Fig. 1). In total, 382 patients were excluded because of previous hydrocephalus history (333 patients) or who died on the same day of admission to emergency department or of hospitalization (51 patients). The remaining 23,775 patients were divided into TBI-S group (2303 patients) and TBI-NS group (21,472 patients) to analyze the risk of PTH in the 2-year follow-up period.

### 3.2 Baseline demographic characteristics of the study patients

The TBI-S group was significantly older, had less monthly income, higher CCI score, and more comorbidities (ie, hypertension, congestive heart failure, diabetes mellitus, stroke, coronary artery disease, atrial fibrillation, and spontaneous SAH) than the TBI-NS group. After propensity score matching, the TBI-S and TBI-NS groups had similar distributions of the baseline covariates and comorbidities. The demographic characteristics of TBI-S and TBI-NS groups in the full cohort and the propensity matched cohort were presented in Table 1.

**Table 1:**
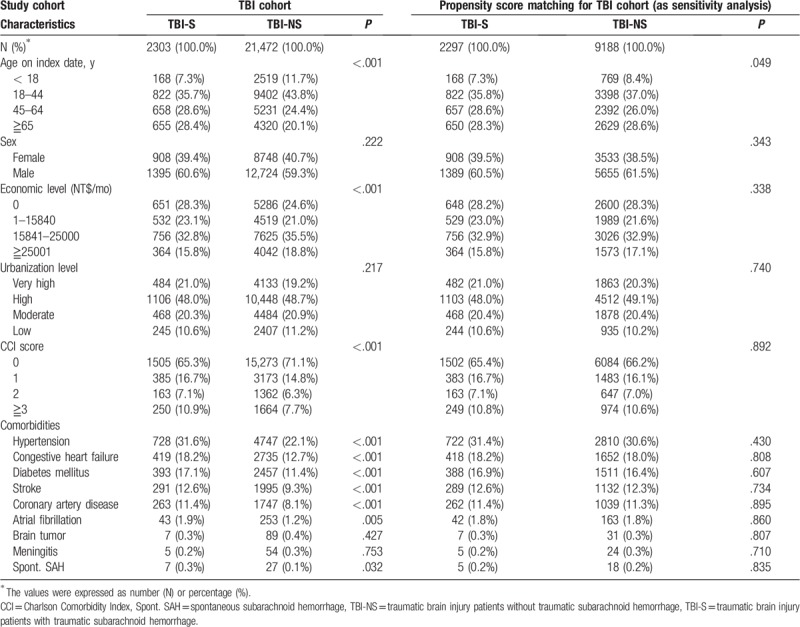
The demographic characteristics of two groups, 2000 to 2010.

### 3.3 The cumulative incidence, incidence rate of PTH

**Figure 2:**
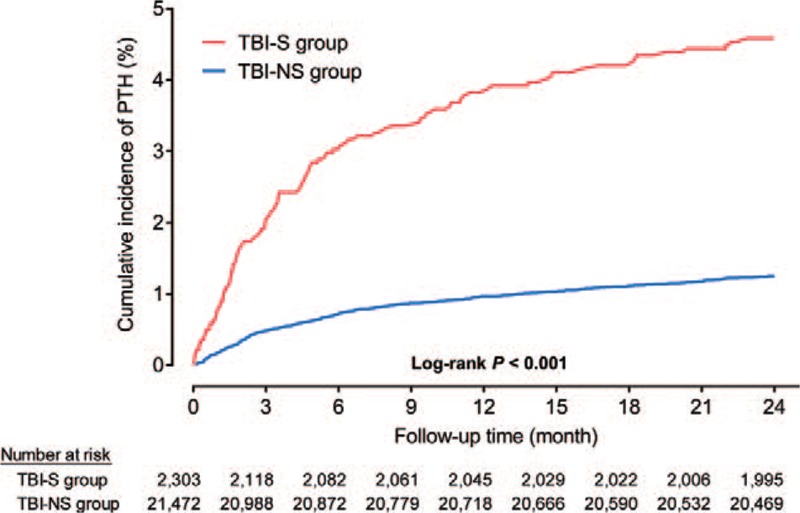
The cumulative incidence of post-traumatic hydrocephalus. Note: Log-rank test was used for comparison between 2 groups. PTH = post-traumatic hydrocephalus, TBI-NS = traumatic brain injury patients without traumatic subarachnoid hemorrhage, TBI-S = traumatic brain injury patients with traumatic subarachnoid hemorrhage.

**Figure 3:**
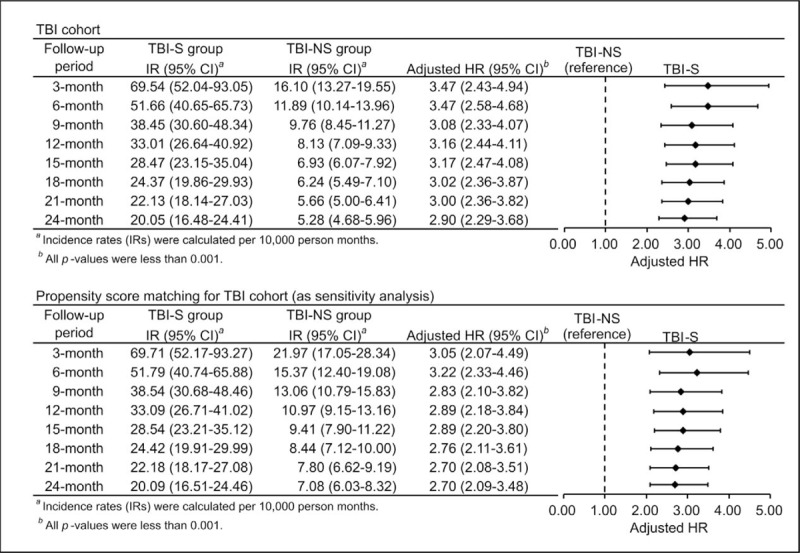
The incidence rate and hazard ratio of post-traumatic hydrocephalus during different follow-up periods in 2 cohort analysis. Note: The hazard ratios were estimated by competing risk regression adjusted for age, sex, urbanization, economic level, Charlson Comorbidity Index score, spontaneous subarachnoid hemorrhage, stroke, meningitis, brain tumor, hypertension, diabetes mellitus, atrial fibrillation, congestive heart failure and coronary artery disease. HR = hazard ratio, IR = incidence rate, PTH = post-traumatic hydrocephalus, TBI = traumatic brain injury, TBI-NS = traumatic brain injury patients without traumatic subarachnoid hemorrhage, TBI-S = traumatic brain injury patients with traumatic subarachnoid hemorrhage.

Figure 2 shows the cumulative incidence of PTH in TBI-S and TBI-NS groups within 2 years after brain injury. Comparing to TBI-NS group, there was a significantly higher cumulative incidence of PTH in the TBI-S group during each of the follow-up periods (Log-rank test: *P* < 0.001, Fig. 2). The incidence rates for TBI-S group were 69.54, 51.66, 33.01, and 20.05 PTH cases per 10,000 person months during 3-, 6-, 12-, 24-month follow-up periods, respectively (Fig. 3). The incidence rates for TBI-NS group were 16.10, 11.89, 8.13, and 5.28 PTH cases per 10,000 person months respectively (Fig. 3).

### 3.4 The HRs of PTH in TBI-S group compared to the TBI-NS group

To investigate the relative risk of PTH in the TBI-S group, compared to those in the TBI-NS group, the HR of developing PTH was calculated for both Cohort 1 and Cohort 2 (Fig. 3). The adjusted HRs of developing PTH in the TBI-S group within 2 years were 2.90 to 3.47, and the highest estimate was obtained within 6 months’ follow-up in the full cohort. Similar results were also found in the sensitivity analysis.

### 3.5 The new event of PTH at different follow-up periods

**Figure 4:**
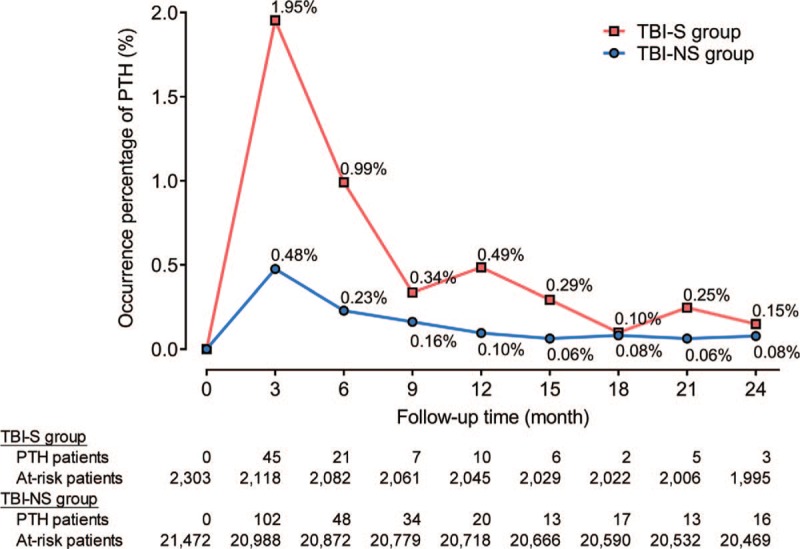
The occurrence percentages of post-traumatic hydrocephalus for 2 groups within every 3 months of 2-year follow-up period. PTH = post-traumatic hydrocephalus, TBI-NS = traumatic brain injury patients without traumatic subarachnoid hemorrhage, TBI-S = traumatic brain injury patients with traumatic subarachnoid hemorrhage.

New events of PTH in TBI-S and TBI-NS groups, expressed as the occurrence percentages within each of the 3-month follow-up periods are shown in Figure 4. The highest occurrence percentage of PTH in both groups developed during 0 to 3rd month. The occurrence percentage of PTH was 1.95% (45/2303) in the TBI-S group and 0.48% (102/21,472) in the TBI-NS group. During the 3rd to 6th month, the percentage of PTH in the TBI-S group decreased to 0.99% and then fluctuated within 0.10% to0.49% during the 9th to 24th month. In the TBI-NS group, the percentage of PTH was fluctuated within 0.06% to 0.23% during the 6th to 24th month.

## 4 Discussion

Our study is to our knowledge the first population-based cohort study to demonstrate a higher risk of PTH in the TBI-S group, when compared to the TBI-NS group. There are 3 key findings in our study. First, we found that the new onset of PTH after TBI was highest during the first 0- to 3rd-month follow-up period in both the TBI-S and the TBI-NS groups. Second, there was a significantly higher cumulative incidence of PTH in the TBI-S group during each of the follow-up periods, when compared to TBI-NS group. Third, the TBI-S group was approximately 3 times more likely to develop PTH than the TBI-NS group.

### 4.1 The development and peak time of PTH

Previous studies variably reported that hydrocephalus post TBI occurred in approximately 0.7% to 45% of affected individuals.^[7,8]^ In our analysis, we found that a new event of PTH affected 1.95% in the TBI-S group and 0.48% in the TBI-NS group during the 0 to 3rd month. The differences in reported rates from existing literature and our study might be related to variations in sample size, inclusion criteria, exclusion criteria, and measurement of hydrocephalus between studies.^[7–14]^ Some previous results from single hospital-based sample^[9,12,13]^ or special subgroups of brain injury^[12–14]^ may lead to selection bias. Toward estimating the incidence of PTH in TBI patient in our country, we performed this largesample, population-based cohort study. 23,775 first event TBI patients requiring hospitalization for ongoing monitor and management were enrolled in our study. The peak time of PTH occurred during 0 to 3rd month in both TBI-NS and TBI-S group. Our study is the first study to provide information on PTH development expressed in terms of cumulative incidence, incidence rate, occurrence percentage in TBI patients with or without traumatic SAH. We hope that the development of PTH expressed in different ways in this study will inform future research.

### 4.2 Traumatic SAH increased risk of PTH

We found that TBI patients with traumatic SAH had an approximate 3-fold risk of developing PTH, comparing to TBI patients without traumatic SAH in the 2-year follow-up period. Although PTH after traumatic SAH has been reported since the 19th century, there have been relatively few studies on risk factors or predictors of PTH after TBI.^[9,18,19]^ It remains uncertain whether traumatic SAH is a risk associated with PTH.^[9,18,19]^ In Kammersgaard et al's study, they found that age, severe disability and low level of consciousness on admission were independent predictors of developing PTH during rehabilitation.^[19]^ However, the presence of traumatic SAH was not associated with PTH onset.^[19]^ In their study, only patients transferred to rehabilitation were enrolled indicating a greater severity.^[19]^ Tian et al's study found that the distribution and thickness of traumatic SAH increased risk of PTH.^[18]^ There was no association however between hydrocephalus and the location of traumatic SAH.^[18]^ A study by Jiao et al noted that patients with traumatic SAH was 43.42-fold risk of developing PTH.^[9]^

In studies which document a higher risk of PTH after traumatic SAH, the risk of PTH was also variable. In our study, the adjusted HR of PTH in the TBI-S group in each 3-month follow-up period of 2 years was between 2.90 and 3.47. These values were lower than those in Tian et al's and Jiao et al's studies.^[9,18]^ The possible reasons may be related to different study designs in different studies. In Jiao et al's study, only patients who met their criteria of severe TBI were included and those whose survival time was <6 months were excluded.^[9]^ Similarly, patients who died during the course of analysis were also excluded in Tian et al's study.^[18]^ In our study, all TBI patients who admitted to hospitalization were included from our population-based dataset. Only patients who had previous TBI, previous hydrocephalus, or who died on the index date were excluded. The remaining TBI patients, ranging from mild to severe injury, were follow-up no more than 24 months or till death. If the patient died during the 2-year follow-up period, this patient was not excluded from our study but was treated as competing event against PTH. Thus, comparing to those in Jiao et al's study and Tian et al's study, our sample may have a greater proportion with lesser severity of injury but our sample size was larger. Because of these different inclusion criteria, exclusion criteria, and statistic analysis among these studies, it leads to wide range of risk estimates of developing PTH after traumatic SAH.

### 4.3 The mechanism of hydrocephalus development

In the traditional theory, the mass effect of blood clots and the obstruction by inflammation-mediated adhesions within the ventricle were the main causes of hydrocephalus.^[1,6,16]^ In recent literature, some researches provided other mechanisms of hydrocephalus development in TBI and SAH model.^[32,33]^

Xiong et al also demonstrated that a dramatic decrease in cilia and CSF flow occurs in the ventricular system in the early phase of the mild TBI mice model.^[32]^ CSF was accumulated in the ventricular system and acute hydrocephalus occurred at this period.^[32]^ Gradually, cilia density was restored to uninjured level by ependymal cell ciliogenesis 30 days after brain injury.^[32]^ This study provided the mechanism of poor CSF reabsorption in acute phase of mild TBI with PTH, especially when there were no initial blood clots in the ventricle system.

In other animal SAH model, Aydin et al found the number of water vesicles of the choroid plexus was affected by neuron density of the petrous ganglion of glossopharyngeal nerve following SAH.^[33]^ In the early phase of SAH, the glossopharyngeal nerve was irritated and leaded to the choroid plexus increase vesicles formation in the ventricular system.^[33]^ The overproduction of CSF eventually developed hydrocephalus.^[33]^ In the late phase of SAH, the petrous ganglion was ischemic and the effect of parasympathetic nerve of the choroid plexus leaded to decrease the water vesicles of the choroid plexus.^[33]^ This study provided the neural controlled mechanism of overproduction of CSF in acute hydrocephalus either in traumatic or nontraumatic SAH cases.

These 2 animal models provided us new concepts of acute hydrocephalus formation in TBI, traumatic or nontraumatic SAH. It explained why the peak time of PTH occurred mainly within 6 months after TBI and then gradually decreased in our study.

### 4.4 Limitations

The main strength of our study is the nationwide population-based cohort sample to investigate the development of PTH within 2 years after traumatic SAH. Potential recall bias and selection bias were thus reduced. However, there were several important limitations, arising from the use of a large cross-national database. First, some potential confounders, such as the characteristics (severity and location) of TBI and SAH, type of surgical intervention, and ventriculitis, were not available. Second, the link between PTH and SAH was extracted from the dataset with a limited follow-up period of 2 years. Thus, the results cannot be generalized to longer-term effects. Future studies to evaluate the underlying mechanism between PTH and traumatic SAH are needed.

## 5 Conclusions

In this study we have demonstrated that the TBI-S group had an approximate 3-fold risk of developing PTH when compared to the TBI-NS group. The peak time of PTH for both TBI-S and TBI-NS groups occurred during the 0 to 3rd month after head injury. Practitioners should pay more attention during the critical period.

## Acknowledgment

The authors are grateful for the support of the Health Information and Epidemiology Laboratory (CLRPG6G0041, CLRPG6G0043) of Chang Gung Memorial Hospital, Chiayi, Taiwan, since 2016. It provided this study a longitudinal health insurance database for analysis.

## Author contributions

**Conceptualization:** Kai-Hua Chen, Chuan-Pin Lee, Yao-Hsu Yang, Yun-Hsuan Yang, Chien-Min Chen, Vincent Chin-Hung Chen.

**Data curation:** Kai-Hua Chen, Chuan-Pin Lee, Yao-Hsu Yang, Vincent Chin-Hung Chen.

**Formal analysis:** Chuan-Pin Lee, Yao-Hsu Yang, Vincent Chin-Hung Chen.

**Methodology:** Chuan-Pin Lee, Yao-Hsu Yang, Vincent Chin-Hung Chen.

**Resources:** Chuan-Pin Lee.

**Software:** Chuan-Pin Lee.

**Supervision:** Vincent Chin-Hung Chen.

**Writing – original draft:** Kai-Hua Chen, Chuan-Pin Lee, Yao-Hsu Yang, Yun-Hsuan Yang, Chien-Min Chen, Mong-Liang Lu, Yi-Chen Lee, Vincent Chin-Hung Chen.

**Writing – review & editing:** Kai-Hua Chen, Chuan-Pin Lee, Yao-Hsu Yang, Yun-Hsuan Yang, Chien-Min Chen, Mong-Liang Lu, Yi-Chen Lee, Vincent Chin-Hung Chen.

Kai-Hua Chen orcid: 0000-0002-8527-8620.
